# Clopidogrel-induced acquired haemophilia A in a post-TAVR patient: a case report and literature review

**DOI:** 10.1093/ehjcr/ytaf459

**Published:** 2025-09-23

**Authors:** Muhammad Zaman, Zonghao Pan, Raaghvi Kohli, Amna Amir, Yani Zhang

**Affiliations:** Department of Internal Medicine, Conemaugh Health System, Johnstown, PA 15905, USA; Department of Internal Medicine, Conemaugh Health System, Johnstown, PA 15905, USA; Department of Internal Medicine, Conemaugh Health System, Johnstown, PA 15905, USA; Department of Internal Medicine, Conemaugh Health System, Johnstown, PA 15905, USA; Department of Internal Medicine, MedStar Washington Hospital Center, Washington, DC 20010, USA

**Keywords:** Acquired haemophilia A, Antiplatelets, Clopidogrel, Ticagrelor, Transcatheter aortic valve replacement, Case report

## Abstract

**Background:**

Acquired haemophilia A is a rare bleeding disorder characterized by spontaneous bleeding. Apart from diagnosing and treating it promptly, finding the causative factor for acquired haemophilia A, if any, is equally important.

**Case summary:**

In this case report, we discuss a 63-year-old female who underwent a recent transcatheter aortic valve replacement and presented to the emergency department with posterior thigh pain, where she was found to have a haematoma. She was ultimately diagnosed with acquired haemophilia A secondary to clopidogrel. In addition to discontinuing clopidogrel, the patient received immunosuppressive therapy, including cyclosporine, prednisone, intravenous immunoglobulins, rituximab, and recombinant factor VIIa.

**Discussion:**

Clopidogrel, one of the most commonly used medications in cardiology, is recognized as one of the leading drugs associated with acquired haemophilia A. In this case report, a review of the literature on ticagrelor and clopidogrel-induced acquired haemophilia A is also included, emphasizing the need for extreme vigilance in patients on antiplatelet therapy who present with unexplained bleeding.

Learning points

- Acquired haemophilia A constitutes a rare but acknowledged adverse effect associated with specific medications.
- Clopidogrel, one of the most commonly used medications in cardiology, is recognized as one of the leading drugs associated with acquired haemophilia A. It is crucial for cardiologists to be aware of this specific effect.

## Introduction

Acquired haemophilia A (AHA) is a rare bleeding disorder characterized by autoantibodies against Factor VIII (FVIII). The aetiology is frequently idiopathic but may be linked to various autoimmune disorders, including systemic lupus erythematosus, rheumatoid arthritis, Sjögren's syndrome, lymphoproliferative malignancies, gestation, and certain pharmacological agents, particularly clopidogrel- an extensively utilized medication in cardiovascular care. The predominant cause of mortality among patients with acquired haemophilia is haemorrhage, and the standard treatment protocol typically entails immunosuppression alongside the management of bleeding episodes. This article presents a case report regarding clopidogrel-associated AHA and examines analogous cases of AHA associated with antiplatelet therapy documented in the existing literature.

## Summary figure

**Figure:**
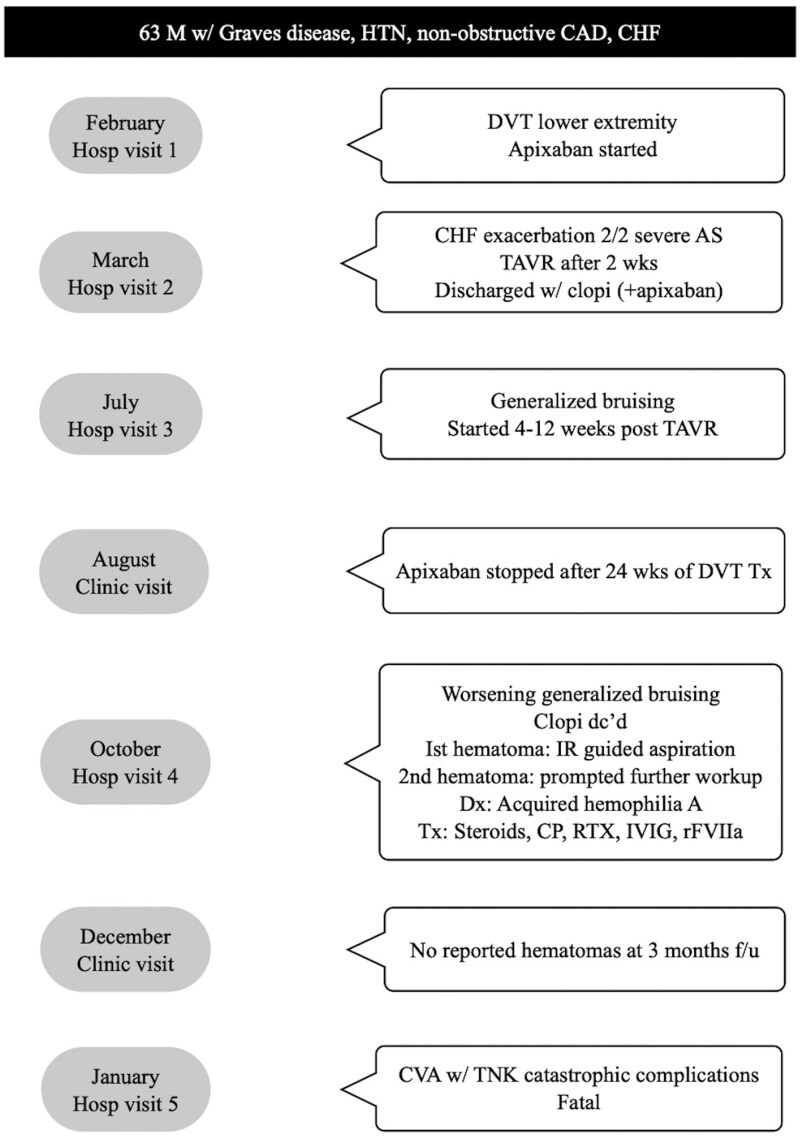


## Case description

A 63-year-old female with a history of Grave’s disease, hypertension, right lower extremity distal deep vein thrombosis (DVT), presented to the hospital with generalized bruising and pain on her posterior left thigh. The patient’s medications included clopidogrel, empagliflozin, rosuvastatin, sacubitril-valsartan, spironolactone, bumetanide, and levothyroxine. Upon initial assessment, her vital signs revealed a temperature of 98.6°F, heart rate of 68 beats per minute, respiratory rate of 16 breaths per minute, and blood pressure of 113/55 mmHg, with oxygen saturation recorded at 99% on room air. The patient reported no incidents of recent trauma or falls.

Five months prior, the patient underwent a transcatheter aortic valve replacement (TAVR) for severe aortic stenosis and congestive heart failure. The procedure was uneventful. She was on apixaban for a remote history of distal DVT, and clopidogrel was added on discharge. Subsequently, she began to experience progressively worsening skin bruising. One month later, she presented with extensive bruising on the left side of her abdomen and chest wall, occurring without any preceding trauma. In the emergency department, her activated partial thromboplastin time (APTT) was elevated at 65 s; the computed tomography (CT) findings were unremarkable, and apixaban was subsequently discontinued.

**Figure 1:**
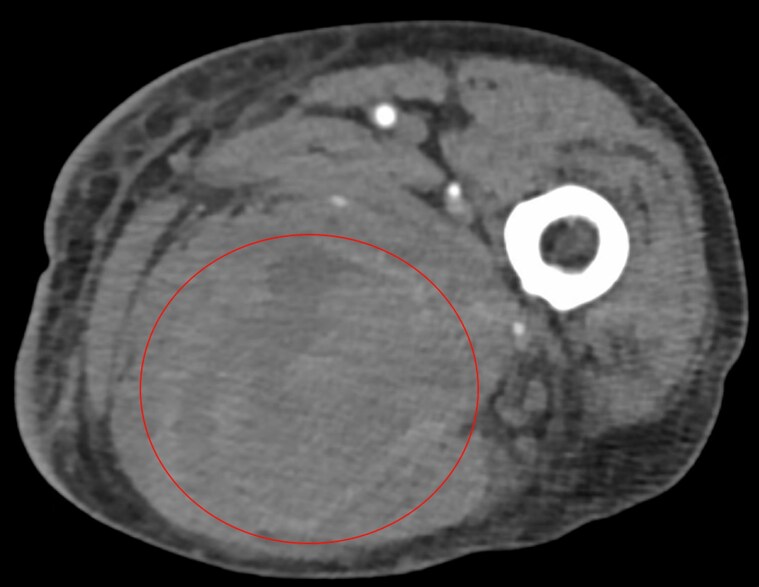
The CT angiographic scan of the left lower extremity reveals the haematoma encircled by the red circle.

The preliminary laboratory results at the presentation indicated a haemoglobin level of 6.8 g/dL and a potassium level of 2.8 mEq/L. The electrocardiogram demonstrated only sporadic premature atrial contractions. The CT angiographic examination of the lower extremities revealed a haematoma measuring 10.3 × 8.7 × 4.5 cm in the left thigh, as depicted in *Figure 1*. The echocardiogram exhibited normal systolic function, grade III diastolic dysfunction, and a normal bioprosthetic valve without perivalvular leakage.

Despite the cessation of clopidogrel for five days, the patient conveyed experiencing severe and persistent pain in the thigh. An aspiration of the haematoma was executed, yielding 50 mL of blood, which subsequently resulted in considerable bleeding at the site of insertion. Furthermore, the patient was administered four units of packed red blood cells. Throughout the hospital stay, the patient exhibited the development of multiple minor bruises and swelling in the posterior region of the left arm, which raised apprehensions regarding the possibility of an additional haematoma. Consequently, a comprehensive haematoma-specific evaluation was undertaken, as detailed in *Table 1*. The APTT mixing study demonstrated a correction indicative of a deficiency in one or more Factors VIII (FVIII), IX, XI, or XII. After the transfusion of one unit of Fresh Frozen Plasma (FFP), it was observed that the activities of Factors XI and VIII were at 36% and consistently below 1%, respectively.

**Table 1:** Specific laboratory workup for aetiology of haematoma evaluation

Laboratory Tests	Reference Range	Patient’s Value	Status
Prothrombin time	9.1–12.0 s	10.3 s	NL
International normalized Ratio	0.9–1.2	0.9	NL
APTT	22.9–30.2 s	59.8 s	High
Thrombin time	0.0–23.0 s	14.3 s	NL
Factor VII activity	50–150%	65%	NL
Factor VIII activity	57–163%	< 1%	Low
Factor VIII:C	0.60–1.50 U/mL	0.02 U/mL	Low
Anti-factor VIII:C	≤ 0.4 BU/mL	144.7 BU/mL	High
DRVVT screen	≤ 47.0 s	41.3 s	NL
vWF antigen	50–200%	197%	NL
vWF multimer analysis	—	Normal pattern and distribution	NL
Factor IX activity	60–177%	128%	NL
Factor XI activity	60–150%	36%	Low
Peripheral blood smear:	—	macrocytic anaemia	
Lupus anticoagulant	—	1.24	weakly positive
Fibrinogen	200–400 mg/dL	446 mg/dL	NL
D-dimer	< 0.5 µg/mL	8.39 µg/mL	NL

APTT, Activated prothrombin time; BU, Bethesda units; DRVVT, Dilute Russell’s viper venom test; INR, International Normalized Ratio; NL, normal; sec, seconds; vWF, von Willebrand factor

The patient received a diagnosis of AHA attributed to the presence of an FVIII inhibitor. An initiation of treatment with cyclosporine at a dosage of 2 mg/kg per day, prednisone at 1 mg/kg per day, and intravenous immunoglobulins at 400 mg/kg over five days was conducted. This therapeutic regimen was subsequently complemented with four weekly administrations of rituximab and six doses of recombinant Factor VIIa. Following a hospitalization duration of one month, the patient was discharged with prescriptions for cyclophosphamide at a daily dosage of 100 mg and prednisone at 50 mg daily. Four months post-discharge, the patient exhibited acute aphasia indicative of a stroke. Without intracranial large vessel occlusion on CT angiography, tenecteplase was administered after a discussion with family. Unfortunately, this intervention was complicated by fatal intracranial and subarachnoid haemorrhages.

## Discussion

AHA is an uncommon autoimmune disorder characterized by immunoglobulin G autoantibodies targeting Factor VIII. The clinical presentation of AHA ranges from asymptomatic instances that necessitate no haemostatic intervention to critical life-threatening scenarios.^1^ Spontaneous bleeding may manifest in the gastrointestinal tract subcutaneously following minor trauma or, albeit rarely, as intracranial haemorrhage.^1,2^ Approximately fifty per cent of AHA cases are classified as idiopathic; the remaining cases are often associated with various factors, including the postpartum phase, autoimmune disorders, malignancies, infectious processes, vaccinations, or pharmacological agents.^3^ An investigation utilizing the World Health Organization's pharmacovigilance database, which analysed 185 cases, identified alemtuzumab, clopidogrel, and omalizumab as the three medications exhibiting the most pronounced pharmacovigilance signal for drug-induced AHA.^4^ The diagnosis of AHA is predicated upon clinical manifestation and an isolated prolongation of activated partial thromboplastin time (APTT) that is not rectified by a 1:1 mixing study. In contrast to alloantibodies in severe haemophilia A, which typically lead to complete inactivation of Factor VIII activity, autoantibodies present in AHA result in only partial inactivation of Factor VIII activity.^5^ As a result, the *in vitro* inhibitor titre may not accurately reflect the *in vivo* inhibitor potency, and neither residual Factor VIII activity nor inhibitor titres correlate with the severity or responsiveness of haemorrhagic events.^1,6^

The foundation of haemostatic management encompasses replacement therapy utilizing recombinant porcine FVIII, bypassing therapy through activated prothrombin complex concentrate (PCC), or recombinant Factor VIIa.^1^ Corticosteroid monotherapy, combined with cyclophosphamide or rituximab, is advisable as the first-line immunosuppressive treatment to eradicate inhibitors.^1^ Corticosteroids facilitate remission in 60% to 90% of cases, with the duration of treatment ranging from a few days to several months.^7^ The prognosis for patients diagnosed with drug-induced haemophilia A is generally favourable, with a complete remission rate of 83.3% attainable following the cessation of the implicated drug or after immunosuppressive therapy.^6^

Clopidogrel, an antiplatelet agent widely utilized in clinical practice, has received approval from the FDA for the management of acute coronary syndrome, cerebrovascular events, and peripheral artery disease, as well as for off-label applications in carotid artery disease and post-TAVR thromboprophylaxis. The active metabolite of clopidogrel irreversibly binds to the P2Y12 adenosine diphosphate receptors on platelets, thus inhibiting platelet aggregation. It is important to note that clopidogrel may result in minor or major haemorrhagic events, particularly among elderly patients or those concurrently using other antithrombotic agents. We undertook a comprehensive literature search using the databases PubMed, Web of Science, Embase, and Scopus, employing the keywords ‘clopidogrel’ and ‘acquired haemophilia,’ ‘ticagrelor’ and ‘acquired haemophilia,’ and ‘prasugrel’ and ‘acquired haemophilia.’ Our search yielded nine case reports of AHA induced by clopidogrel and two cases associated with ticagrelor; however, no instances involving prasugrel were found. These cases and the present case are systematically summarized in *Table 2*.

**Table 2:** Case reports of similar cases in the literature

Author	Age/Sex	Presentation	Medication	Indication	P2Y12 Duration	APTT (seconds)	FVIII Level	Anti-FVIII Inhibitor	Treatment	Remission
Haj *et al*., 2004^8^	70 F	excessive bruising and soft tissue bleeding	clopidogrel	PAD	2–3 months	48.6	3.9 IU/dl	2.2 BU	prednisolone	FVIII inhibitor undetectable in 8 weeks
	67 F					77.6	1 IU/dl	17.6 BU		
Shimizu *et al*., 2019^9^	80 M	subcutaneous haemorrhage	aspirin, clopidogrel	PTA for PAD	10 months	prolonged	—	449 BU	oral corticosteroid therapy	APTT normalized in 4 weeks
Hwang *et al*., 2012^10^	65 M	blood oozing from tracheostomy site	aspirin, clopidogrel	cerebellar infarction	3 weeks	40.3 → 98.8	<1%	5.4 BU/mL	steroids	APTT normalized, FVIII inhibitor undetectable in a few days; FVIII 8% with bleeding at 3 weeks; FVIII normalized at 2 months with repeat steroids
Liu *et al*., 2024^11^	73 M	severe anaemia, suspected GI bleeding	aspirin, clopidogrel	cerebral infarction	3 years	89.7	1.0%	>8.0 BU	declined treatment	—
Ali *et al*., 2016^12^	87 M	ecchymoses, left iliacus haematoma after fall	clopidogrel	PTA for PAD	4 months	60	4%	—	corticosteroids, IVIG and rFVIII, FVIIa	APTT normalized after 3 weeks
Donath *et al*., 2022^13^	72 F	epistaxis, > 20 duodenal/jejunal bleeding AVM, an intramural haematoma	aspirin, clopidogrel	PCI w/DES	2 months	85	<1%	>30 BU	prednisone, rituximab, rFVIII	—
Qolbi *et al*., 2021^14^	60 M	haemarthrosis, anaemia, epistaxis	aspirin, clopidogrel	PCI w/DES	—	102.2	—	—	methylprednisolone, tranexamic acid	APTT improved to 59 after 8 days
Zaman *et al*., 2025^15^	63 F	bruising, spontaneous intramuscular haematoma	clopidogrel	TAVR	4 months	59.8	0.02 U/mL (<1%)	144.7 BU/mL	prednisone, cyclophosphamide, IVIG, rituximab, rFVIIa	—
Vartak *et al*., 2024^16^	68 M	RP bleeding, haematomas in the iliopsoas, chest wall, and eye orbit	aspirin, ticagrelor	PCI w/DES	8 days	95.5	<1%	144 BU	Steroids, rFVIIa, FEIBA	—
Pasquino *et al*., 2016^17^	52 M	right leg pain, haemorrhagic effusion	aspirin, ticagrelor	PCI w/DES	1 month	80	1.1%	6.72 BU	prednisone, cyclophosphamide with continuation of DAPT	FVIII level normalized in few weeks, inhibitor titre was negative

Abbreviations: APTT, activated partial thromboplastin time; IU, international unit; BU, Bethesda units; DAPT, dual antiplatelet therapy; PAD, peripheral arterial disease; PCI, percutaneous coronary intervention; PTA, percutaneous transluminal angioplasty; TAVR, transcatheter aortic valve replacement; RP, retroperitoneal; DES, drug-eluting stents; FEIBA, Factor VIII inhibitor bypassing activity; IVIG, intravenous immunoglobulin; rFVIIa, recombinant activated Factor VII; rFVIII, recombinant Factor VIII; FVIIa, activated Factor VII

AHA associated with ticagrelor typically manifests sooner (between 8 and 30 days) than clopidogrel, which can take 21 days to 10 months for presentation. Among the 11 documented cases, three resulted in mortality, and two were complicated by cerebrovascular accidents potentially associated with the administration of recombinant coagulation factor. Dermal ecchymosis is prevalent, yet it is often attributed to the antiplatelet effects of clopidogrel, resulting in the oversight of coagulation studies and a delayed or missed diagnosis of AHA. Nevertheless, an abnormal APTT is often the earliest and most significant laboratory indicator, warranting immediate assessment for AHA. Prompt recognition and commencement of corticosteroid-based immunosuppressive therapy are vital and potentially life-saving.

This case underscores the relationship between clopidogrel and acquired haemophilia, accentuating the significance of timely diagnosis and intervention to avert life-threatening complications. Given the ambiguous pathogenesis and the infrequency of clopidogrel-associated acquired haemophilia, comprehending its clinical manifestations is of utmost importance, particularly from a cardiologist's perspective. Misattributing haemorrhagic episodes solely to clopidogrel may obscure the diagnosis of drug-induced AHA, thereby postponing timely treatment.

## Data Availability

The data underlying this article are available in the article. Additional data underlying this article will be shared at reasonable request to the corresponding author.

## Lead author biography

Muhammad Adnan Zman is a post graduate internal medicine resident in US. His interests include cardiovascular medicine with focus on hyperlipidaemia, temporary mechanical support devices for cardiogenic shocks.
